# The Origin and Evolution of the 18th Century Hospital Movement
*Previous articles appeared on December 13, 1913, January 17 and 31, February 14 and 28, and March 14 and 28, 1914.


**Published:** 1914-04-11

**Authors:** 


### VIII. THE ORIGIN AND EVOLUTION OF THE 18th CENTURY HOSPITAL MOVEMENT * (*concluded*).

It is said that until the erection of the Hospital for Smallpox and Inoculation in 1746 no institution existed in the whole of Europe for the reception and treatment of this disease, which was then credited with a mortality ten times greater than that of any other affection, in the absence of plague. Its widespread dissemination, the need of immediate isolation and treatment, and the fact that the doors of all hospitals were closed against it by prohibitive by-laws, were urged as additional reasons for such an institution, though, not unexpectedly, the scheme met with considerable opposition. The practice of inoculation, which had been introduced twenty years earlier by Lady Mary Wortley Montagu, was unpopular in spite of statistics asserting that, whereas the case-mortality of those who contracted the disease in the natural way was nearly 20 per cent., only one person out of 150 had died after inoculation. The disease itself was regarded with the utmost fear and abhorrence, and the inhabitants of Cavendish Square and the district surrounding the hospital in Windmill Street complained of its propinquity as a public nuisance. Removal from one place to another was invariably followed by opposition, until, premises having been purchased in Coldbath Fields, the legal authorities decided in favour of the charity and an injunction was refused by Lord Chancellor Hardwicke.

### The Erection of the Lock Hospital.

Scarcely had it commenced a career marked by more vicissitudes than any other charity, than the hospital movement found further encouragement in the erection of the Lock Hospital, the memory of whose chapel, once a fashionable place of worship like that of the Foundling, is preserved by the name of Chapel Street (Grosvenor Place). It was said that persons suffering from venereal disease were more destitute of relief than any other objects of public charity, for being excluded from the county hospitals, and the treatment in the country districts being "often uncertain and sometimes fatal," they were obliged to resort to London for relief. From early times the orders of the chartered hospitals had excluded such cases from all but the two Lock out-houses belonging to St. Bartholomew's, while the by-laws of all other hospitals, except the London, for long afterwards denied them admission, and though the governors of the chartered institutions "out of prudent compassion had made some further provision for them" by erecting "foul" wards, many patients were still refused for want of room, and others, unable to find the securities, fees, and contingencies, remained unrelieved, became incurable and perished.†

### The First Hospital for Unmarried Mothers.

The lying-in ward which had been set apart by the governors of the Middlesex Infirmary in 1747 had been the means of provoking dissensions among the subscribers, some of whom complained that contributions given for that special purpose had been diverted to general uses. Foiled in their disingenuous attempt to change its constitution into that of a lying-in charity (there being no hospital, except in Dublin, solely employed for that purpose, and the workhouse infirmaries being the only receptacles for such cases), the dissenting subscribers established in 1749 the Lying-in Hospital for Married Women in Brownlow Street, Long Acre, this site being chosen as midway between the cities of London and Westminster. In the following year the City of London Lying-in Hospital was founded to supply the wants of the former locality, and in 1752 the General Lying-in Hospital, afterwards known as Queen Charlotte's, was instituted in Westminster, this latter being the first charity of its kind to receive unmarried women.

### The Early "Madhouses."

The insufficiency of the accommodation at Bedlam is said to have been the cause of the establishment of St. Luke's Hospital for the Insane in 1751; but although both of them were situated in Moorfields they were totally distinct charities, the former being one of the Royal foundations with charter and endowment, the latter supported entirely by voluntary contributions.

In addition to these two public institutions for "lunatics" and the cells which were provided in the workhouses for incurable cases, numerous private madhouses existed for the reception of the wealthier classes; but until 1774 there was no legal direction for their supervision, and even then the Act did not apply to the poor, who were sent to asylums without any authority beyond that of their parish officers.

### Dean Swift as a Hospital Founder.

St. Patrick's Hospital in Dublin, founded and built by the bequest of Dean Swift for the reception of lunatics and idiots, was incorporated in 1746, but was not opened for patients until 1757.

The latter half of the eighteenth century was characterised by the spread of the hospital movement to the Midland and Northern Counties, and in five instances to Scotland; but Wales does not seem to have been provided with any public hospital until those established at Denbigh in 1807, and Swansea in 1817. In Ireland very few institutions existed for the reception of the sick poor outside Dublin, until it was ordered by an Act of the Irish Parliament, 5 Geo. III. (1765), that an infirmary should be erected in every county, some of which appear to have been established upon existing foundations. These were supported by grants, assessed by the grand jury of the county in which each was situated.

In London, no further general hospitals were established until the Charing Cross in 1818, which was followed by University College Hospital in 1833, and St. Mary's in 1845.

### The Dispensary Movement.

The long interval of quiescence is accounted for to some extent by the movement for the establishment of dispensaries, which resulted in the abstraction of many subscriptions and legacies from the voluntary hospitals, whose position was thereby seriously jeopardised. The first of these was the General Dispensary opened in Aldersgate Street in 1770, which was followed in a few years by similar institutions in every quarter of London. But whereas in the original dispensary scheme of the College of Physicians patients were left to procure at

---

* Previous articles appeared on December 13, 1913, January 17 and 31, February 14 and 28, and March 14 and 28, 1914.

† The first Lock Hospital in Ireland was founded by Mr. Doyle, a Dublin surgeon, and was opened for the reception of women and children in 1755, and of male patients in 1758.

their own cost the medicines prescribed, which many were unable to do, these later institutions, supported by voluntary subscriptions, following the design of the Westminster Charitable Society, administered advice, medicine, and attendance when necessary at the patient's home, free of all expense. They thus afforded relief to the mother who could not leave her family, to the infant who could not be parted from its mother, and to the chronic asthmatic and consumptive who, in the crowded state of the hospitals, could not find admission. Before the close of the eighteenth century many of these dispensaries had been established in London, and scarcely a provincial town of any size was without at least one such institution, which in course of time was frequently converted into an infirmary or hospital.

From Hospitality to Treatment.

In pre-Reformation days each religious house possessed its infirmary for the sick. Many of these religious houses were called hospitals, connoting the hospitality offered to their guests. The idea of medical treatment was subordinate to the food and entertainment. After the suppression of the religious houses the infirmaries which they had contained were neither reconstituted nor replaced. Of the five Royal hospitals, only two made provision for the sick; and these became associated in the public mind, and in the popular speech, with the administration of medical and surgical aid. The idea of food and entertainment had become subordinate to the treatment of disease. The poor who met with accident or illness went to the hospital for cure.

When workhouses arose in the early years of the eighteenth century, quarters were set apart as infirmaries for the sick; and, as time went on, the infirmary became a separate building. The early voluntary institutions at Westminster, Cork, and Edinburgh were all styled infirmaries; the French Protestant and Guy's, which assimilated in many respects to the Royal foundations, were called hospitals. In modern times the Poor Law, and most of the provincial, medical institutions have the monopoly of the title of infirmary; all the large London charities are hospitals.

Henry Hoare, Edmund Burke, and John Howard.

In tracing the history of the hospital movement of the early part of the eighteenth century, prominence has been given to the benevolence of Henry Hoare; and as we approach its close the labours of John Howard are not forgotten. "I cannot name this gentleman," said Edmund Burke, "without remarking that his labours and writing have done much to open the eyes and hearts of mankind. He has visited all Europe—not to survey the sumptuousness of palaces, or the stateliness of temples; not to make accurate measurements of the remains of ancient grandeur, not to form a scale of the curiosity of modern art; not to collect medals, or to collate manuscripts—but to dive into the depths of dungeons; to plunge into the infection of hospitals; to survey the mansions of sorrow and pain; to take the gauge and dimensions of misery, depression, and contempt; to remember the forgotten, to attend to the neglected, to visit the forsaken, and to compare and collate the distresses of all men in all countries. It was a voyage of discovery; a circumnavigation of charity. Already the benefit of his labour is felt more or less in every country; I hope he will anticipate his final reward, by seeing all its effects fully realised in his own" (*Burke's Speech at the Guildhall, Bristol*, 1870).

